# Human Cystic Echinococcosis: Old Problems and New Perspectives

**DOI:** 10.1155/2009/474368

**Published:** 2009-11-01

**Authors:** Alessandra Siracusano, Antonella Teggi, Elena Ortona

**Affiliations:** ^1^Dipartimento di Malattie Infettive, Parassitarie e Immunomediate, Istituto Superiore di Sanità, Viale Regina Elena 299, 00161 Roma, Italy; ^2^Dipartimento di Malattie Infettive e Tropicali, Ospedale Sant'Andrea, Università di Roma, “Sapienza”, 00189 Roma, Italy

## Abstract

Cystic echinococcosis (CE) is a widespread chronic endemic helminthic disease caused by infection with metacestodes of the tapeworm *Echinococcus granulosus*. CE affects humans and has a worldwide prevalence of approximately six million. In this review, we discuss current findings in diagnosis and clinical management of CE and new concepts relating to *E. granulosus* molecules that directly modulate the host immune responses favouring a strong anti-inflammatory response and perpetuating parasite survival in the host. New insights into the molecular biology of *E. granulosus* will improve considerably our knowledge of the disease and will provide new potential therapeutic applications to treat or prevent inflammatory immune-mediated disease.

## 1. Introduction

Although effective preventive and therapeutic measures have been developed for most parasitic helminths, cystic echinococcosis (CE) infection is still very common in the developing world today. CE, a widely chronic endemic helminthic disease caused by infection with metacestodes (larval stage) of the tapeworm *Echinococcus granulosus*, is one of the most widespread Helminth zoonotic diseases in humans [1–5]. *E. granulosus* metacestode infections are characterized by the development of slowly growing hydatid cysts which may not be detected for months or years after the initial infection has occurred. The persistence of these cysts is of interest to immunologists since, once fully formed, they are apparently unaffected by the hosts immune response. The host parasite relationship is interactive and the outcome of infection depends on the balance achieved by the combination of the different variables involved with the host immunity and the parasite avoidance strategies. An understanding of the biological events occurring during infection is necessary to visualize the diverse immune stimuli to which the host is subjected and to define diagnostic and therapeutic tools. The evidence suggests that the intermediate hosts respond independently to antigenic stimuli of the invading oncosphere, the metacestode in transformation from the oncosphere, and finally, the mature metacestode (larvae) [6].

The oncospheres hatch and become activated in the small intestine when a suitable intermediate host ingests *Echinococcus* eggs. Lytic secretions of the oncosphere then facilitate its passage through the intestinal mucosa and into the host circulatory system (venous and lymphatic) through which they are distributed to the liver, lungs, and other sites where postoncospheral development continues. Within a few days after, oncospheres reach the preferred site, cystic development begins. This process involves degeneration of the oncospheral stage and emergence of the metacestode stage. In vitro 4–7 days are required for larvae to change into a typical “bladder” with a germinal layer. Subsequently, development time varies widely from one host species to another. In general, hydatid cysts increase in diameter from less than 1 to 5 cm each year. The mature hydatid cyst consists of an inner germinal layer of cells supported externally by an acellular-laminated membrane of variable thickness. Tegumental cells of the germinal layer unite to form a continuous syncytium which is differentiated into numerous microvilli that project peripherally into the laminar layer toward the host tissues surrounding the hydatid cyst. Small secondary cysts called “brood capsules” bud internally from the germinative layer and, by asexual reproduction, produce multiple protoscoleces that can differentiate either into adult worms in the intestines of definitive hosts or into secondary hydatid cysts following rupture of a cyst in the intermediate hosts.

Because the oncosphere is known to be associated with the protective immune response, understanding the mechanisms, whereby protective antibodies against the oncoshere act, is of fundamental importance in developing highly effective vaccine against *E. granulosus* [6]. A vaccine based on the recombinant oncosphere protein, Eg95, has been produced for prevention of infection in the parasite's natural animal intermediate hosts [7, 8].

## 2. Clinical Aspects: Diagnosis and Therapy

Despite the advances in modern imaging and therapeutic strategies, problems associated with the diagnosis and treatments of human CE are still challenging and often difficult to resolve. Early diagnosis is important, because prompt intervention enables efficient management and treatment of the disease and results in reduced morbidity and mortality. Initially, cysts are small and patients are asymptomatic [9, 10]. Cysts in such instances are too small, too young, to pose clinical problems; usually they are single and localized in a neutral space in liver or lungs. The cysts may heal spontaneously by inconspicuous rupture and evacuation, or by degenerative and/or necrotic processes leading to a solidification and/or calcification of the cysts. Apart from lung cysts, which are more often symptomatic also if they are very small, symptomatic cases of CE are usually those with the cyst's diameter >5 cm. In most cases initially, the clinical diagnosis of CE is difficult and requires, beside the physical examination, imaging techniques and serology [11, 12]. As the cysts grow, however, they can exert mechanical pressure on surrounding organs and can cause several pathological changes mediated by compression or obstruction. Large amounts of hydatid fluid, after cyst rupture, can result in anaphylactic reaction that varies widely from benign urticaria and short episodes of shaking chills and/or fever, to a potentially fatal bronchial spasm, angioneurotic oedema, and anaphylactic shock. Cyst rupture can also result in a secondary hydatid infection caused by the release of many thousands of protoscoleces, which each have the potential to differentiate into another hydatid cyst.

Introduction of ultrasound (US) in the late 1970s has greatly improved the detection of liver and abdominal *Echinococcus* cysts and differential diagnosis from common nonparasitic true cysts and pseudocysts of traumatic, degenerative, inflammatory, and malignant origin. In particular, this noninvasive, low-risk, and low-cost examination can also have a positive educational effect in endemic communities [13]. The combination of US and confirmatory serology is now a standard approach for clinical and epidemiological surveys [14]. Whereas hepatic cysts can be preferentially diagnosed by US, hydatid cysts localized in lungs or in other organs than liver and abdomen can be successfully diagnosed by computerized tomography (CT) or Nuclear Magnetic Resonance imaging (NMR). In 2001, the WHO Informal Working group on Echinococcosis proposed an international classification of hepatic cysts based on US morphology correlated to the activity of the disease [10, 15]. Following the WHO classification, hepatic hydatid cysts are grouped into five major cyst types, CE1-CE5, characterized by the appearance of the cyst contents and wall. Using such classification has enabled clinicians to examine recommended clinical procedures for the different cyst types. If “Cystic Lesions” are defined as suspicious lesions, and if due to CE, then these cysts are usually at an early stage of development and are not fertile. Type CE1 (unilocular, simple cysts) and type CE2 (multivescicular, multiseptated cysts) are considered as active since they are likely to contain viable protoscoleces. Type CE3 (unilocular cysts with detachment of laminated membrane or multiseptated cysts with partial hyperechoic content) are considered as transitional and might represent the beginnings of cyst degeneration. Type CE4 (heterogeneous or hyperechoic degenerative contents) and type CE5 (calcified cysts) are considered inactive, as the parasite tissue is likely to be of low viability. Actually, we can consider the current WHO classification as a progressive natural history of cyst development from CE1 to CE5. It is of huge importance to understand the possible developmental fate of a cyst, but US imaging alone cannot predict the clinical fate of a cyst. Cysts classified by US as identical in clinical type may have distinctly differing fates: some will ultimately progress whereas others will regress (mainly, types CE3 and CE4). New interesting observation on the immunological mechanisms underlying these distinct outcomes indicate that the serologic profile associated with cysts of the same ultrasonographic type (type CE 3-4-5) correlates with the fate of the cyst: higher IgG1 and IgG3 in stable disease and higher IgG4 and IgE in progressive disease [16]. Because the advanced diagnostic techniques used today, for example, ultrasound scanning, detect even young (early stage) cysts (less than 2 cm in diameter) and calcified (late stage) cysts, providing more sensitive serological tests which are still a pressing research aim. In fact, a key problem for immunodiagnosis is the increasing numbers of seronegative patients or low responders, who still have too few antibodies detectable by serologic tests. To overcome problems of cross-reactions with other parasite antigens in countries with endemic disease, it is important to have new more antigens that are specific. The correct diagnosis of CE involves clinical, laboratory, and epidemiological information. As far as possible, alternative diagnostic methods are obligatory for suspected CE images in the US [10]. In fact, without confirmation of immunological tests, clinical and/or imaging technique diagnosis is not certain, unless for histological and/or cytological examination, being CE cysts hardly distinguished from “space occupying lesions” [5, 17]. Immunodiagnosis is useful not only for the primary diagnosis but also for the follow-up of patients after surgical or pharmacological treatment, or both [10, 17]. Hydatid serological testing has a long history and almost all serological tests that have been developed have been used in the diagnosis of human cases. The choice of a serodiagnostic technique depends primarily on its sensitivity and specificity. The first problem is that most conventional tests give a high percentage of false-negative results (up to 25%). Secondly, false-positive reactions are present in areas where *E. granulosus* and *E. multilocularis* coexist and in areas where other parasitic diseases are endemic. An optimum test should be specific with high sensitivity. Immunodiagnostic techniques include initial screening tests, using crude antigens, such as latex agglutination, double diffusion, indirect hemagglutination, and enzyme-linked immunosorbent assay, followed by confirmatory tests using specific antigens, for example, arc-5 immunoelectrophoresis and immunoblotting [11, 12]. In particular, immunoblotting technique uses antibodies to identify specific target proteins and is now widely employed in routine laboratory applications for the analysis of immune responses [5]. Another diagnostic strategy for identifying active or current infections is to develop a technique for detecting circulating antigen in serum and urine [18, 19].

The history of immunodiagnosis represents the history of *Echinococcus* antigens because the choice of an appropriate source of antigenic material is a crucial point in the immunodiagnosis [5]. As the intermediate host of *E. granulosus,* humans are exposed to a variety of antigenic determinants on parasite-derived or parasite-modified molecules; each of these various sources of antigenic stimulation may be relevant to immunodiagnosis. The repertoire of antigens to which the host is exposed includes many factors, for example, the species or strain of parasite, host immunocompetence, organ parasitised, cyst fertility, cyst viability, and integrity of the cyst wall [20]. Antigens derived from *Echinococcus* oncospheres have not yet been successfully adapted to immunodiagnostic tests; however, such antigens provide theoretical advantages over metacestode stage antigens for a variety of purposes, including early detection of infection.

Extensive studies have focused on hydatid fluid antigens that still represent the main antigenic source for hydatid disease diagnosis. At the present time, the parasitic antigens present in hydatid fluid that have major immunodiagnostic value in detecting *E. granulosus *are antigen 5 (Ag5) and antigen B (AgB) [21]. Native Ag5, a 400 kDa thermolabile lipoprotein produces two subunits at 55 and 65 kDa in sodium-dodecyl sulphate-polyacrilamide gel electrophoresis (SDS-PAGE) under nonreducing conditions and two subunits at 38/39 and 20 kDa under reducing conditions [22, 23]. The 38/39 kDa component with phosphorylcholine epitopes may be responsible for a large proportion of cross-reactions with sera from patients infected with nematodes, cestodes, and trematodes [24–26]. Native AgB, a 160 kDa thermostable lipoprotein, produces three main subunits at 8/12, 16, and 20 kDa in SDS-PAGE under reducing and nonreducing conditions as well as other mass-subunits, probably polymers of the 8/12 kDa subunit [27]. The 8/12 kDa subunit induces a good humoral and cellular response [28]. Even though the 8/12 kDa subunit of AgB is cross-reactive in a high-percentage patients with alveolar echinococcosis sera and in a small percentage of patients with cysticercosis, native AgB is of high immunodiagnostic value [21, 24, 29].

To overcome some problems several recombinant antigens have been produced and used as molecular tools in the immunodiagnosis of CE [5, 30]. Although a few studies have assessed the diagnostic usefulness of recombinant Ag5 intense research has evaluated recombinant AgB and synthetic peptides that could be used in immunodiagnosis of CE [30–32]. To date, the only peptide that has proved diagnostic is that derives from the sequence of EgAgB1p176 [33]. Although native and recombinant antigens yield similar sensitivity, both have advantages and disadvantages [31]. Native antigens have to be extracted from the various sources, purified and prepared for use in each laboratory. They are, therefore, of limited availability, and nonstandardized in quality and composition. Recombinant antigens, usually commercially prepared and then distributed to the various diagnostic centres, have the advantage of being standardized in quality and composition.

For many years, surgery has been considered the only treatment available for CE because of the potential radical removing of the parasite. Nowadays, besides surgery, clinical management of CE may relies on several therapeutic approaches ranging from chemotherapy with benzoimidazole carbamates (mebendazole and albendazole) to percutaneous nonconventional treatment, like PAIR, or Radio-Frequency Thermoablation, or “wait and see” approach [9]. New guidelines on clinical management of liver CE suggest that there is not a single “golden rule” but it is very important to individualize the treatment of choice for each patient and for each hydatid cyst [9, 13, 34, 35]. A series of early articles have comprehensively reviewed the clinical management of CE [9, 13, 36, 37]. Here, we consider the more recent progress in the clinical management during hepatic involvement of CE. In the therapeutic approach to liver CE, it is advisable to distinguish the cases in which there is a concordance between clinical and serological data and imaging findings, and the cases in which these findings are discordant. In the first cases, and for no complicated small hepatic cysts (of diameter <5 cm, CE4-CE5 type), it is possible to take into account the “wait and see” approach because inactive cysts that do not compromise organ function or cause discomfort, seem to remain like this or stabilize even further. When clinical, immunological data and US findings are not concordant or when the cysts are complicated, surgery represents the first choice treatment [9, 13]. Surgery is indicated for large hepatic cysts with multiple daughter cysts; for single hepatic cysts, situated superficially, which may rupture spontaneously, or because of trauma; for cysts that are infected; for cysts communicating with biliary tree and/or exerting pressure on adjacent vital organs. Because curative surgery is not always possible, there is a 2–15% risk of relapse in hyperendemic areas and moderate ranges of morbidity in particular when the surgery is repeated. During surgery, the cyst can break spontaneously or the surgical damage of the cyst can lead to spillage and widespread dissemination [9, 36, 37].

Chemotherapy with benzoimidazole carbamates (mebendazole or albendazole), once reserved for inoperable cases of CE, is now more widely used [34, 37–41]. Benzamidazole carbamates inhibit tubulin and induce blockage of glucose absorption, glycogen depletion, and degenerative modifications in the endoplasmatic reticulum and in mitochondria of the germinal layer increasing lysosomes and producing cellular autolysis [41]. Mebendazole and albendazole have the same mechanism of action; albendazole shows a better pharmacokinetic profile reaching higher plasmatic concentrations than mebendazole. At present, a cycle of albendazole treatment can be suggested as first choice treatment in patients with no complicated cysts and in patients without contraindications to chemotherapy (pregnancy, marked impairment of liver, renal or haemopoietic functionality). Most of the cysts treated with benzoimidazole carbamates show degenerative modifications (volumetric reduction and/or morphological alterations, such as solidification, detaching of membranes, and calcification) whose further evolution can hardly be predicted: in some cysts these degenerative modifications progressed until the parasite's death (biological recovery), while some cysts recurred after the end of treatment. Apart quantitative differences due to a more effectiveness of albendazole for hepatic cysts, albendazole, and mebendazole show similar effects. Treatment with benzoimidazole carbamates is effective and well tolerated but can be affected by many factors related both to the host and to the parasite. Both albendazole and mebendazole are more effective in cycles of continuous treatment, without intervals. It is relevant to note that young cysts and cysts of young people are more responsive, probably because these cysts present a higher metabolic activity and a greater susceptibility to the drugs. Benzoimidazole carbamates are more effective against cysts in the lung than against cysts in the liver that may be because of their thinner membranes. Regarding the type of hepatic cysts, CE1 cysts frequently show membrane detachment after treatment, while CE2 cysts frequently show matrix solidification. Sometimes cysts of the same patient with the same morphology and localized in the same organ may differently respond to therapy probably because have a different intrinsic sensitivity to drugs. Some treated patients' exhibit relapses, but these are usually sensitive to retreatment in high proportion of cases (up to 90%) [42]. Chemotherapy with benzoimidazole carbamates appears to be safe and well tolerated, the main adverse events are to changes in transaminases (<5-fold the normal range), observed in about 15% of the patients. To note, this side-effect is reversible, because transaminase value returned to normal without stopping treatment and it was mainly observed in patients with hepatic cysts and effectiveness of therapy [37, 43]. The increase in transaminases may be caused by the inflammatory immune response to the antigenic spillage from the hepatic cysts damaged by benzimidazole carbamates, and because correlates with the effectiveness of therapy could be considered as a prognostic marker. This finding further supports the importance of immune-mediate mechanisms in the clinical outcome of chemotherapy of CE, as observed for antibiotic treatment and innate and cell-mediated immunity. Because there is no doubt that, as an adjuvant therapy, chemotherapy can significantly contribute to the successful management of CE, the search for new drugs is ongoing [44].

In the last two decades, the percutaneous treatment by Puncture of the cyst, Aspiration of cyst fluid, Injection of a scolicidal agent, and reaspiration of the cyst content percutaneous (PAIR) under sonographic guidance has gained an important role in the treatment of CE; its efficacy has been confirmed both by short- and long-term follow-up. The aim of this treatment is to destroy the germinal layers with scolicidal agents or to evacuate the germinal and laminated layers, that is, the entire endocyst [45]. Percutaneous drainage of echinococcal cysts is effective and safe, as shown by the very low complication rate. Because neither imaging modalities nor serology is sufficient to assess directly the presence of parasites in the cyst, PAIR is the only method providing a direct diagnosis of the parasitic nature of the cysts. The major risks of percutaneous techniques are anaphylactic shock, secondary echinococcosis caused by spillage of cystic fluid, and chemical colangitis caused by contact of the scolicidal agent with the biliary tree.

Radiofrequency thermal ablation uses the same needle electrodes used for local treatments or hepatocellular carcinomas [46]. The experience with radiofrequency thermal ablation is still very limited; however, it does not seem to be very effective at long-term follow-up [13].

## 3. New Promising Perspectives from the Host-Parasite Relationship

Evidence from epidemiological studies indicates an inverse correlation between the incidence of certain immune-mediated diseases, including inflammatory bowel disease, and exposure to Helminth. Helminth parasites are the classic inducers of anti-inflammatory Th2 responses. Cross-regulatory suppression of the Th1 responses by a strong Th2 response has a role in modulating diseases characterized by a Th1 response. In particular, the Th2-polarized T cell response driven by Helminth infection correlates with the attenuation of some damaging Th1-driven inflammatory responses, preventing some Th1-mediated autoimmune diseases in the host [47–49].

Current evidence concerning antibody levels of IgG4 and IgE isotypes and frequent eosinophilia in CE suggests that the immune response to established *E. granulosus* infection is Th2 dominated and that *Echinococcus* antigens modulate polarized T-cells. These observations received confirmation from studies showing that the human immune response to *E. granulosus *infection is predominantly regulated by Th2 cell activation (in vitro production of IL-4, IL-5, IL-6, IL-10 by PBMC isolated from patients with CE) and also by the Th1 (or Th0) cell subset (IFN-*γ* production) [50]. Many findings indicated that in CE a strong Th2 response correlates with susceptibility to disease (active cyst), whereas a Th1 response correlates with protective immunity (inactive cyst) and that Th1 and Th2 responses coexist [6, 50, 51].

During CE, the distinguishing feature of the host-parasite relationship is that chronic infection coexists with detectable humoral and cellular responses against the parasite. *E. granulosus *could use two mechanisms to subvert the host immune response: passive escape, in which the parasite, by developing into a hydatid cyst, avoids the damaging effects of an immune response, and immunomodulation, through which the parasite actively interacts with the host immune system to reduce the impact of a host response [12]. Recent studies demonstrated that *E. granulosus* secretes several molecules present in protoscoleces and in hydatid fluid that directly can modulate the immune responses thus altering the cytokine balance towards Th2 and favouring their evasion and perpetuating their survival in the host [52–54]. These molecules interfere with antigen presentation, cell proliferation and activation, antibody production, cause cell death, and stimulate regulatory responses.

The abundance of AgB in hydatid fluid suggests that this antigen has an important biological role in *E. granulosus* infection. AgB is involved in modulating the host immune response altering both innate and adaptative host immune responses. The 12-kDa subunit of AgB is a protease inhibitor that can inhibit neutrophil recruitment and that has a critical role in parasite escape mechanisms from early natural immunity [55]. Investigating further, the role of AgB in the host-parasite relationship has been confirmed that AgB impairs the inflammatory response and influences the Th1/Th2 balance towards a Th2 polarization [56]. To note, *E.* g*ranulosus* AgB acts to escape the host immune response by interfering directly with host dendritic cell function through two strategies [57]. First, it impairs monocyte precursor differentiation into immature dendritic cells rendering them unable to mature when stimulated with LPS; secondly, AgB modulates sentinel dendritic cells maturation, priming them to polarize lymphocytes into Th2 cells.

The possibility that IgG subclasses from patients with active or inactive CE might contain antibodies against molecules involved in the host-parasite interaction has been extensively examined [58]. In vitro AgB driven Th2 cytokine production corresponds in vivo to elevated specific IgE and IgG4 antibody binding to the 8 kDa subunit of AgB [16, 58, 59]. A new immunomodulating antigen has been obtained by screening an *E. granulosus* cDNA library with IgG4 from patients with active disease: a protein localized in the protoscolex tegument and on the germinal layer of cyst wall, named EgTeg. However, EgTeg, similarly to AgB, inhibits chemotaxis and induces IL-4-positive T lymphocytes and noncomplement-fixing antibodies (IgG4) [60].

CE shares with other helmintiases three responses typical of immediate hypersensitivity reactions such as elevated IgE/IgG4 antibodies production, eosinophilia, and mastocitosis, which contribute to trigger a Th2-type environment. By screening an *E. granulosus* cDNA library with IgE from patients with CE with acute cutaneous allergic manifestations have been identified three conserved constitutive proteins: EgEF-1 *β*/*δ*, EA21, and Eg2HSP70 associated with allergic disorders related to CE [61–63].

The hygiene hypothesis arose from attempts to explain differences in allergy prevalence related to socioeconomic and geographical factors. An inverse relationship between helminthiasis and allergy has been clearly established despite both conditions being accompanied by strong Th2 immune responses. [64]. The CE example stresses the ambiguous links that exist between parasitic and allergic diseases, and shows how studying these disease can help to understand how immune deviation leads to pathological events and to find new immunomodulatory or preventive drugs or both [64].

## 4. Concluding Remarks and Future Directions

Despite the large efforts that have been put into the research and control of echinococcosis, it still remains a disease of worldwide significance. In some areas of the world, CE caused by *E. granulosus* is a re-emerging disease in places where it was previously at low levels. Although ultrasound images and benzoimidazole carbamates are very useful in the clinical treatment of CE, achieving complete healing of the infection require defining more clearly the immunological events that accompany changes in cyst morphology.

Exposure to Helminths may protect from immune-mediated diseases and this evidence suggests that Helminths may have served as a lid on a “Pandora's box” of immune pathology. *E. granulosus* has evolved to live within its mammalian host, and in order to do so appear to express a diverse array of molecules that have immune-modulating effects. These observations suggest that *E. granulosus* molecules could be used therapeutically to treat or prevent immune-mediated disease.

Future studies understanding the mechanisms of *E. granulosus* immune regulation, will potentially uncover novel compounds that alter inflammatory responses, and will address the myriad of questions surrounding their potential for clinical application.
